# Chronic Stress and Alzheimer’s Disease-Like Pathogenesis in a Rat Model: Prevention by Nicotine

**DOI:** 10.2174/157015911798376307

**Published:** 2011-12

**Authors:** Karim A Alkadhi

**Affiliations:** Department of Pharmacological and Pharmaceutical Sciences, College of Pharmacy, University of Houston, Houston, TX 77204, USA

**Keywords:** Rat AD model, amyloid-beta, learning and memory, signaling molecules, synaptic plasticity, chronic nicotine, chronic stress.

## Abstract

Environmental factors including chronic stress may play a critical role in the manifestation of Alzheimer’s disease (AD).This review summarizes our studies of the aggravation of the impaired cognitive ability and its cellular and molecular correlates by chronic psychosocial stress and prevention by nicotine in an Aβ rat model of AD. We utilized three approaches: learning and memory tests in the radial arm water maze, electrophysiological recordings of the cellular correlates of memory, long-term potentiation (LTP) and long-term depression (LTD), in anesthetized rats, and immunoblot analysis of synaptic plasticity- and cognition-related signaling molecules. The Aβ rat model, representing the sporadic form of established AD, was induced by continuous i.c.v. infusion of a pathogenic dose of Aβ peptides *via* a 14- day osmotic pump. In this AD model, chronic stress intensified cognitive deficits, accentuated the disruption of signaling molecules levels and produced greater depression of LTP than what was seen with Aβ infusion alone. Chronic treatment with nicotine was highly efficient in preventing the effects of Aβ infusion and the exacerbating impact of chronic stress. Possible mechanisms for the effect of chronic stress are discussed.

## 1 INTRODUCTION

Alzheimer’s disease (AD) is an irreversible, progressive neurodegenerative brain disorder characterized by extra-cellular accumulation of pathogenic amyloid-beta (Aβ) peptides, intracellular aggregation of hyperphosphorylated tau protein, and neuronal death [1, 2]. The early symptom of the disease is a slow and insidious destruction of memory and cognitive skills. Molecular studies have shown that missense mutations in genes for amyloid precursor protein (APP), presenilin 1 (PS1) or presenilin 2 (PS2) account for the majority of familial AD cases [3-5]. However the early-onset familial AD represents less than 5% of AD cases, while the sporadic, late-onset AD is evident in the vast majority of the cases [4, 5]. Because of the poor correlation of the severity of dementia with the extent of neuronal loss and degree of fibrillar Aβ load in the AD brain, the original amyloid cascade hypothesis has been substantially revised. It is now thought that certain oligomeric forms of soluble Aβ can cause cognitive impairment in animals in the absence of neuro-degeneration [6]. Additionally, synaptic plasticity, including long-term potentiation (LTP) and long-term depression (LTD), the cellular correlates of learning and memory, are highly vulnerable to disruption by soluble Aβ species [7].

The sporadic nature of this disorder suggests an environmental link that may trigger AD pathogenesis. In addition to its late-onset, the variation in susceptibility to and time of onset of the disease suggests that aside from genetic factors, environmental determinants, such as chronic stress, may also play a critical role in the severity of sporadic form of AD. Additionally, during AD, a progressive failure of synaptic transmission occurs; it begins as a localized decrease in synaptic function, and over time, progresses to global impairment of neurotransmission in the brain [4, 7, 8].

Chronic stress is a homeostatic challenge with physical and psychological ramifications and a particularly negative effect on the learning and memory process [9-14]. It is known that stress aggravates cognitive impairment in various disorders including schizophrenia [15], Cushing’s disease [16], hypothyroidism [17], and AD [18-21]. Based on clinical reports of elevated plasma cortisol levels in individuals with dementia and in AD patients [22-25], it has been postulated that stress may be associated with this disease [26-28]. Further support of this hypothesis comes from epidemiological findings that stressed individuals are more likely to develop mild cognitive impairment, or even AD, than non-stressed individuals [29, 30]. Clinical reports of hypercortism in AD patients [25, 31] and animal studies [32, 33] have shown that glucocorticoids participate in the regulation of APP levels suggesting involvement of these hormones in the pathogenesis of AD. Stress activates the hypothalamic-pituitary-adrenal (HPA) axis resulting in glucocorticoids blood levels high enough to activate type-II glucocorticoid receptors with negative consequences for hippocampal function [34-36]. Because of the abundance of glucocorticoid receptors in the hippocampus and its involvement in cognition, chronic stress can have deleterious effects on the hippocampal structure and function [37].

Most epidemiological studies have reported a highly significant negative correlation between cigarette smoking and AD [38-40, 42 but see 42, 43]. Laboratory and clinical studies have shown that nicotine improves cognitive function in AD patients and attenuates Aβ-induced amnesia in rodents [19, 40, 44, 45].The finding that chronic nicotine treatment prevents stress-induced down regulation of central nicotinic acetylcholine receptors (nAChRs) [46], suggests a mechanism by which nicotine may prevent stress-induced impairment of memory and LTP. Moreover, the observation that stress-induced atrophy of hippocampal neurons reversibly impairs cognitive function, suggests that chronic nicotine treatment may reduce the negative impact of excitotoxic amino acids and corticosteroids, and subsequently, prevent permanent damage and cognitive decline.

The combined effects of psychosocial stress and nicotine in AD have not been studied thoroughly in any AD animal model; hence this review summarizes recent findings that are largely reported from this laboratory.

## 2 THE Aβ RAT MODEL OF AD

A number of neuropathological features of AD have been reproduced in mice by the introduction of APP, PS1, and PS2 transgenes [47-51]. The majority of transgenic mice exhibit cognitive deficits, amyloid peptides accumulation, and synaptic dysfunction, without showing neurofibrillary tangle formation, neuronal death, or microglial activation [52-55]. The establishment of double or triple-transgenic mice has improved the phenotypic similarities between animals and humans [56,57]. However, certain major limitations of transgenic mouse models of AD have been recognized. For example, the cerebrospinal fluid of these AD models contains a constant, high concentration of various Aβ peptides, thus complicating investigation of the molecular bases of synaptic dysfunction. Additionally, the lack of neuronal death suggests that compensatory factors may be triggered by the introduction of transgenes into these mice [58].

As a complementary alternative to transgenic animal models, non-transgenic models of AD are valuable tools for studying the specific pathogenesis induced by Aβ. Similar to transgenic models, exogenous Aβ administration does not reproduce the full complexity of the human AD pathology. However, studies involving exogenous administration of Aβ have reported neurodegeneration and microglial activation, proximal to Aβ deposits [50, 60]. Exogenous Aβ administration model of AD is not without limitations. For example, injection/infusion of Aβ peptides is an invasive procedure, particularly when using osmotic pumps. The injury at the site of infusion may contribute to the induction of inflammatory processes. However, these limitations can be overcome to a significant degree by adjusting the infusion rate, the vehicle, the volume of injection, and the recovery time.

During normal cellular metabolism, neurons secrete low levels of soluble Aβ peptides into cerebrospinal fluid and plasma [61, 62]. These peptides and their precursor, APP may have a physiological role in synaptic structure and function [63]. It has been suggested that the extent and rate at which the pool of soluble Aβ oligomers accumulates is dependent on the rates of Aβ catabolism and clearance [62, 64-66]. The Aβ rat model was established by continuous osmotic pump infusion of a mixture of Aβ_1-40_ and Aβ_1-42_(300 pmol/day) for 14 days. Control rats were similarly infused with the non-toxic reverse peptideAβ_42-1 _[18-21].

## 3 CHRONIC STRESS INTENSIFIES COGNITIVE DEFICITS 

The radial arm water maze (RAWM) is a hybrid of the radial arm maze and the Morris water maze; it combines the variable spatial complexity of the radial arm maze with the rapid motivated learning of the Morris water maze while minimizing their disadvantages. It is a reliable and sensitive behavioral test for analyzing hippocampus dependent learning and memory [67-69].

Seven experimental groups were designated as control, stress, nicotine, Aβ, nicotine/Aβ, stress/Aβ and nicotine/stress/Aβ. The stress and stress/Aβ groups were subjected to daily stress for 6 weeks and the Aβ and Aβ/stress groups were infused with a mixture of Aβ_1-40_ and Aβ_1-42 _(300 pmol/day) during the fifth and sixth week. The control and stress groups were infused with Aβ_42-1_, an inactive reverse peptide. The RAWM training protocol consisted of a learning phase of four 1-min consecutive learning trials-followed by a short-term and a long-term memory tests, 20 min and 24 hr, respectively, after the last learning trial. The animals had to locate a black platform submerged 1 cm below the water level near the end of one of the 6 swim arms, This procedure was conducted for a minimum of 8 consecutive days or until the rat satisfied a condition called days to criterion (DTC), which is defined as the number of days in which the rat commits a maximum of one error in three consecutive days in the fourth learning trial and memory tests [18-21, 70, 71].

Days 6-8 of testing in the radial arm water maze clearly showed the significantly impaired ability of the stress/Aβ rat group to learn compared to all other groups including the Aβ group. For example, in trial 4, stress/Aβ rats made significantly more errors in locating the hidden platform than the other rat groups including Aβ rats. Furthermore, the Aβ group made significantly more errors than the control and stress rat groups [18-20]. Chronic nicotine treatment (1 mg/kg/12 hr) completely prevented the effect of Aβ and the combination of Aβ and stress [20]. Neither chronic stress alone nor nicotine alone had a significant effect on learning, which is in agreement with our previous findings [72]. The effects on the learning curve were confirmed in the DTC test. In the learning phase, the stress/Aβ rats required approximately twice the number of days as did the control and stress groups to reach the criterion for learning. Fig. (**1A**) [20].

Short-term memory was significantly impaired in both the stress and Aβ groups. However, the stress/Aβ group showed significantly greater impairment of short-term memory than all other groups. These results were further confirmed by the DTC test for short-term memory, which showed that although the Aβ and stress groups required significantly more days to reach the criterion than control, the stress/Aβ group required significantly more days than these two groups Fig. (**1B**) [18]. The deficits in stress, Aβ and stress/Aβ groups were prevented in all nicotine-treated groups (nicotine/stress, nicotine/Aβ and nicotine/stress/Aβ groups [18, 19]).

The 6-week psychosocial stress paradigm impairs short-term memory but does not impair learning or long-term memory in normal rats [18, 73]. However, infusion of Aβ in chronically stressed rats (stress/Aβ group) caused a severe impairment of long-term memory [20] that was significantly greater than that caused by Aβ infusion alone Fig. (**1C**). Again, nicotine prevented the effects of Aβ and stress/Aβ on long-term memory Fig. (**1C**) [20].

## 4 CHRONIC STRESS EXACERBATES IMPAIRMENT OF SYNAPTIC PLASTICITY IN AD MODEL

To relate cognitive deficit to possible changes in the cellular substrate of memory, we evaluated synaptic plasticity in area CA1 of the hippocampus. We recorded population spikes (pSpike) from area CA1 of anesthetized rats and determined changes in the slope of field excitatory postsynaptic potential (fEPSP: a measure of synaptic strength) and pSpike amplitude (a measure of the number of neurons firing action potentials) [72]. In these electro-physiological experiments, we first assessed basal synaptic function by applying a range of stimulus intensities to generate input-output (I/O) curves for all seven groups of animals. The I/O curves of Aβ and stress/Aβ groups showed a significant rightward shift compared with those of control and stress groups indicating impaired basal synaptic transmission in animals infused with Aβ [18]. Chronic treatment with nicotine prevented impairment of basal synaptic transmission in these groups [18].

### 4.1 Early Phase Long-Term Potentiation (E-LTP)

E-LTP is believed to be a cellular correlate of short-term memory [74]. High frequency stimulation (HFS) in control rats induced a robust E-LTP, which lasted up to 3 hrs after HFS. However, in the stress, Aβ and stress/Aβ animals, the maximum increase in the response 5 min after HFS, was only about 50% of that of the control animals Fig. (**2A**) [18]. The E-LTP magnitude, measured as fEPSP or pSpike amplitude, in these 3 groups gradually decayed such that, at 60 min post-HFS the fEPSP slope of the stress/Aβ group was not different from that of the base line, thus, was significantly lower than those of the other groups including the stress and the Aβ groups [18, 19]. In the nicotine treated Aβ and stress/Aβ rats, E-LTP was not significantly different than those of the control or nicotine alone groups Fig. (**2A**).

### 4.2 Late Phase LTP (L-LTP)

L-LTP, evoked by multiple-train HFS, was so severely impaired in the stress/Aβ rats that at 5 hr after HFS, the slope of fEPSP was significantly lower than the baseline Fig. (**2B**) [21]. The magnitude of L-LTP of animals of the Aβ group was significantly lower than that of control group. The magnitude of L-LTP of the three nicotine treated rat groups (nicotine, nicotine/stress/Aβ and nicotine/Aβ) was not significantly different than that of the control group. Thus, nicotine prevented the Aβ-induced impairment of synaptic plasticity. It is interesting to note that although stress markedly accentuates the effect of Aβ infusion, it has no significant effect on L-LTP in normal animals [7] as we have reported earlier Fig. (**2B**) [11, 21, 73, 75].

### 4.3 Long-Term Depression (LTD)

Just as high levels of synaptic activity potentiate synaptic transmission, low levels of persistent stimulation depress hippocampal synapses. Thus, to examine the mechanism responsible for stress and/or Aβ-mediated impairment of LTP, we examined the magnitude of n-methyl-d-aspartate (NMDA)-dependent LTD expressed in the Schaffer collaterals pathway using paired pulse protocol in anesthetized animals [21, 72, 75]. All animal groups displayed robust LTD, which was measured as decreases in the slope of fEPSP. Chronic stress or Aβ- infusion, alone, caused a significantly greater reduction in synaptic strength than that seen in control animals. The magnitude of LTD in stress/Aβ animals was significantly greater than that in control, stress, and Aβ animals. Chronic nicotine administration prevented the effect of stress and/or Aβ on LTD and restored the synaptic signal to a magnitude comparable to that of control animals [21].

## 5. CHRONIC STRESS ACCENTUATES ALTERED LEVELS OF SIGNALING MOLECULES ESSENTIAL FOR MEMORY AND SYNAPTIC PLASTICITY

Calcium calmodulin kinase II (CaMKII) plays a critically important role in the memory and LTP processes. Under normal conditions, induction of LTP by HFS leads to a persistent increase in the levels and activity of phosphorylated (p)-CaMKII and calcineurin in hippocampal slices [76] and anesthetized animal hippocampi [9, 11, 18]. Activation of NMDA receptor causes a transient increases in intracellular calcium concentrations leading to autophosphorylation of CaMKII [77]. The rapid autophosphorylation of CaMKII results in a constitutively active CaMKII [78] that phosphorylates and activates α-amino-3-hydroxy-5-methyl-4-isoxazole (AMPA) receptors and the synaptic vesicle-specific protein, synapsin, which are important for LTP expression [76,79,80]. It is proposed that activation of CaMKII serves as a molecular switch that converts transient Ca^2+ ^signals into long lasting biochemical changes that trigger synaptic plasticity [81].

To further elucidate the potential mechanism by which stress exacerbated Aβ-induced impairment of cognitive abilities and synaptic plasticity, we evaluated the effect of chronic stress and Aβ on the basal levels of signaling molecules essential for these processes by immunoblot analysis. Table **1** summarizes the results of these experiments.

### 5.1 Basal Levels of CaMKII and Calcineurin

Although chronic stress or Aβ markedly reduced the basal levels of phosphorylated CaMKII, these levels were significantly more reduced in the CA1 of stress/Aβ animals Fig. (**3A**) [18]. In normal animals, nicotine did not change the basal levels of this enzyme, but it completely prevented the effect of stress, Aβ or stress/Aβ on the levels of CaMKII Fig. (**3A**) [20].

Phosphorylated CaMKII is normally dephosphorylated by a protein phosphatase, principally calcineurin, which is a negative modulator of cognitive memory and seems to be involved in AD pathogenesis. For example, inhibiting calcineurin with tacrolimus (FK506) reversed cognitive impairment in Tg2576 mice [82]. Immunoblot analysis revealed significant increase in the basal levels of calcineurin in the stress, Aβ and stress/Aβ groups compared to those of the control group Fig (**3B**) [18, 21] and that nicotine treatment normalized calcineurin levels in these animals Fig. (**3B**) [21].

### 5.2 Basal Levels of CREB and CaMKIV

cAMP response element-binding protein (CREB) signaling, a necessary component for hippocampus-dependent long-term memory formation in mammals [83, 84], is severely compromised by Aβ [85]. We measured basal levels of phosphorylated and total (phosphorylated and non-phosphorylated) CREB. Although the basal levels of p-CREB were significantly reduced in the Aβ and stress/Aβ groups, the levels of total CREB were unchanged in these groups [20]. The ratio of basal protein levels of p-CREB to t-CREB was significantly (p<0.05) decreased in Aβ rats, and stress/Aβ rats, compared to control rats Fig. (**4A**) [20]. The decreases in the ratio of p-CREB:t-CREB correlated with reduced phosphorylation of CREB. Furthermore, that the ratio of p-CREB to t-CREB was significantly higher in nicotine/Aβand nicotine/stress/Aβ rats compared to Aβ and stress/Aβ rats, but not significantly different from control rats, suggested that chronic nicotine treatment prevented Aβ- and stress/Aβ-induced inhibition of CREB phosphorylation [20].

During expression of L-LTP, there is an increase in the basal levels of CaMKIV, which directly phosphorylates CREB [86]. We reported that basal levels of CaMKIV were significantly reduced in Aβ animals and stress/Aβ animals, compared to control animals [20]. By contrast, six weeks of chronic nicotine treatment normalized the basal levels of CaMKIV in these two groups. The basal levels of CaMKIV in nicotine, stress, nicotine/Aβ, and nicotine/stress/Aβ animals were not significantly different from control animals [20].

### 5.3 Basal Levels of Brain Derived Neurotropic Factor (BDNF)

The levels of neurotrophic factors, including BDNF, are increased in specific brain regions in response to various types of insults, including ischemia, seizure, traumatic brain injury, and neurotoxins [87]. A possible role for nicotine-mediated neuroprotection has been suggested by studies showing that nicotinic receptor activation increases the levels of neurotrophic factors, including BDNF [88]. Since BDNF plays a major role in neuronal survival [89], we examined the protein expression of BDNF in the seven groups of rats. Immunoblot analyses revealed significantly (p<0.05) higher levels of BDNF in Aβ and stress/Aβ rats, compared to control rats Fig. (**4B**) [20]. The increased basal protein levels of BDNF in area CA1 of Aβ-infused rats is in agreement with an earlier report that shows an increase in the protein levels of BDNF in the forebrain in APPsw mice [90].

That we have detected an increased level of BDNF protein in an area heavily susceptible to Aβ toxic effects is in line with the role of BDNF in the maintenance and repair of neurons [91] and suggests that in early AD, a compensatory mechanism is activated to protect neurons from Aβ-induced neurotoxicity. Furthermore, as chronic nicotine treatment is known to upregulate BDNF protein level and mRNA expression in area CA1 [9, 90], it is not surprising that BDNF protein levels are significantly increased in nicotine, nicotine/Aβ, and nicotine/stress/Aβ rats, compared to control rats Fig. (**4B**). This finding is in agreement with earlier reports showing that chronic nicotine treatment increased BDNF protein levels in APPsw mice [90] and normal rats [9].

We have shown that BDNF levels in stress/Aβ rats are not significantly different from nicotine/stress/Aβ rats [20]. The lack of a significant difference may be due to maximal expression of BDNF in the presence of a chronic disease state. For example, in post-mortem AD brains, BDNF levels have been shown to be significantly higher in the parietal cortex and hippocampus, compared to controls [87]. It is worth mentioning that Hock and colleagues [92] reported a 2-fold decrease in mRNA levels of BDNF in AD postmortem parietal cortex. This suggests that, perhaps, in early stages of AD, BDNF protein levels are increased to bring about protection of neuron from the onslaught of the pathogenic peptides and that at later AD stages, BDNF protein levels are decreased. Together, the data suggest that BDNF is involved in the regulation of nicotine-mediated neuroprotection in Aβ and stress/Aβ rats [20].

## 6 POSSIBLE MECHANISMS OF THE EFFECTS OF STRESS

Previously, we have shown that chronic stress decreases basal levels of p-CaMKII in the CA1 region, and subsequently reduces the magnitude of HFS-induced LTP [11, 19]. Furthermore, the presence of abnormal levels of Aβ peptides disrupts phosphorylation of CaMKII and interferes with LTP induction, as reported in both *in vivo* and *in vitro* studies [19, 93, 94]. Based on findings from our model, we propose that decreasing CaMKII-dependent protein phosphorylation may contribute to the mechanism by which chronic stress impairs memory and LTP in this model of AD.

In general, activation of mineralocorticoid (type-I) receptor by low levels of corticosteroids produces Ca^2+ ^influx, which has an excitatory effect on hippocampal CA1 pyramidal cells, whereas activation of glucocorticoid (type-II) receptor by high levels of corticosteroids during stressful conditions greatly enhances Ca^2+ ^influx and inhibits CA1 pyramidal cell excitability [35, 95]. Given the stress-induced glucocorticoid effects on Ca^2+^ dynamics, it is not surprising that stress worsens Ca^2+^-dependent signaling processes in Aβ rats. This finding is in line with previous reports suggesting that Aβ perturbs intracellular Ca^2+^ signaling [26, 96, 97] and inhibits Ca^2+^-dependent post-translational protein phosphorylation [64]. For example, studies by Zhao *et al.* [94] using acute application of Aβ_1-42 _during HFS showed inhibition of LTP in the dentate gyrus, with corresponding reductions in p-CaMKII levels.

Brain-derived neurotrophic factor plays a major role in neuronal survival [89, 91]. The levels of neurotrophic factors, including BDNF, are increased in specific brain regions in response to various types of insults, including ischemia, seizure, traumatic brain injury, and neurotoxins [87, 98]. Earlier reports that show an increase in the protein levels of BDNF in the forebrain in APPsw mice [90] and area CA1 in Aβ-treated rats [19], suggest that in early AD, a protective mechanism may be activated to counter the Aβ-induced neurotoxicity. In contrast, chronic stress has been reported to significantly decrease BDNF levels in area CA1 of the hippocampus [9]. Therefore, by limiting the availability of BDNF, stress interferes with the repair process and consequently exacerbating the effect of Aβ.

Interestingly, recent reports have shown that the expression of nerve cell adhesion molecule (NCAM) is increased in the brains of AD patients [99] suggesting probable neurogenesis [100]. This could be an attempt by the brain to repair or replace neurons lost to the disease. In contrast to AD, chronic stress is known to cause severe reduction in NCAM levels [100, 101]. We speculate that the neurotoxic effect of Aβ in the brain might be initially offset through repair as suggested by the reported increased levels of NCAM. However, in the presence of chronic stress, the ability of NCAM to repair is severely limited by the stress-induced reduction in the concentration of these protein molecules.

Another possible mechanism for the stress effect is that stress may alter the processing and production of various AD-related proteins. It has been shown that exposure to stress or glucocorticoids increases the levels of APP, C99, and BACE, suggesting that stress is driving the processing of APP toward the amyloidogenic pathway which may account for the increased levels of Aβ [18, 19, 102] and the increased amount of plaque formation [103] that are also observed with stress.

## 7 POSSIBLE MECHANISM OF THE NEUROPROTECTIVE EFFECTS OF NICOTINE

Our finding that nicotine reduces amyloid levels [19] is in accordance with reports that nicotine and its metabolites (cotinine) inhibit β-amyloidosis [104-106]. Based on the original “binding surface hypothesis” of Hilbich *et al.* [107] the authors suggest that nicotine and cotinine delay or inhibit β-amyloidosis by non-specifically binding to Aβ and preventing an α-helix to β-sheet conformational conversion [104-106]. It is postulated that nicotine binds to histidine residues (His6 and His13) on the α-helix [104], or to small, soluble β-sheet aggregates [106], and increases the average separation between Aβ monomers in solution, thus delaying the onset of aggregation [106, 108]. Similarly, studies using nornicotine, another nicotine metabolite, demonstrate that nornicotine-based covalent glycation of lysine-16 on the Aβ peptide, occludes the Aβ polymerization domain, and thus, delays formation of the oligomeric β-sheet structure [105]. It is unclear whether nicotine-mediated inhibition of Aβ deposition, aggregation, and/or α-amyloidosis is due to altered processing (towards a non-amyloidogenic Aβ sequence), decreased synthesis, or increased clearance of Aβ peptides. However, a recent study found that chronic nicotine treatment (1 mg/kg/day and 8 mg/kg/day) reduced rat CSF levels of APPγ, which contains the amyloidogenic Aβ fragment, without significantly altering total soluble APP levels [109, 110]. This suggests that nicotine exerts its effects, in part, by altering the processing of APP away from an amyloidogenic route, towards increased production of APP-carboxyl-terminally truncated forms, which do not contain Aβ_1-40 _and/or Aβ_1-42_. Furthermore, whereas no single mechanism for nicotine-mediated protection has been determined, studies have shown that nicotine increases the levels of neuronal growth factors including BDNF [9, 111, 112], decreases the levels of nitric oxide generated in response to neuronal injury [113], and inhibits glutamate-evoked arachidonic acid release from cultured striatal neurons [114].

Another possible mechanism by which nicotine attenuates the AD-like symptoms is by opposing inflammatory effect of the disease. Inflammatory response is one of the first immune processes in injury. It involves the production of specific molecules that set off the migration of immune cells to wherever the lesion site is, including in the brain. Thus, in stroke, as well as during chronic illnesses, including AD, inflammation takes place in order to clear out and isolate the lesion area. However, sustained inflammation can cause neurotoxicity, which exacerbates the severity of the disease. It is well known that cholinergic anti-inflammatory cascade regulates inflammatory cytokine production through the vagus nerve-dependent pathway involving α7-nicotinic acetylcholine receptor (α7nAChR). Activation of this pathway decreases tumor necrosis factor (TNFα) and blood interleukin-1β levels, inhibits TNF production and reduces pro-inflammatory gene expression. Initiation of this pathway through vagus nerve stimulation or directly by treatment with nicotinic receptor agonists, including nicotine, activates macrophagal α7nAChRs, resulting in anti-inflammatory effects [115-118]. Thus, the α7nAChR-mediated anti-inflammatory action may also contribute to the anti-AD effects of nicotine.

## 8. SUMMARY 

In summary, the presence of chronic stress accentuates the severity of phenotypes in AD rat model. This impairment is likely associated with a number of inter-related disturbances of various signaling molecule pathways including failure of phosphorylated CaMKII to increase after the induction of LTP. The results of these studies suggest that in addition to the onslaught of Aβ-associated cognitive insults wrought on the AD brain, the coincidence of chronic stress further compromises mental abilities in AD patients and accelerates the progression of the disease.

## Figures and Tables

**Fig. (1):**
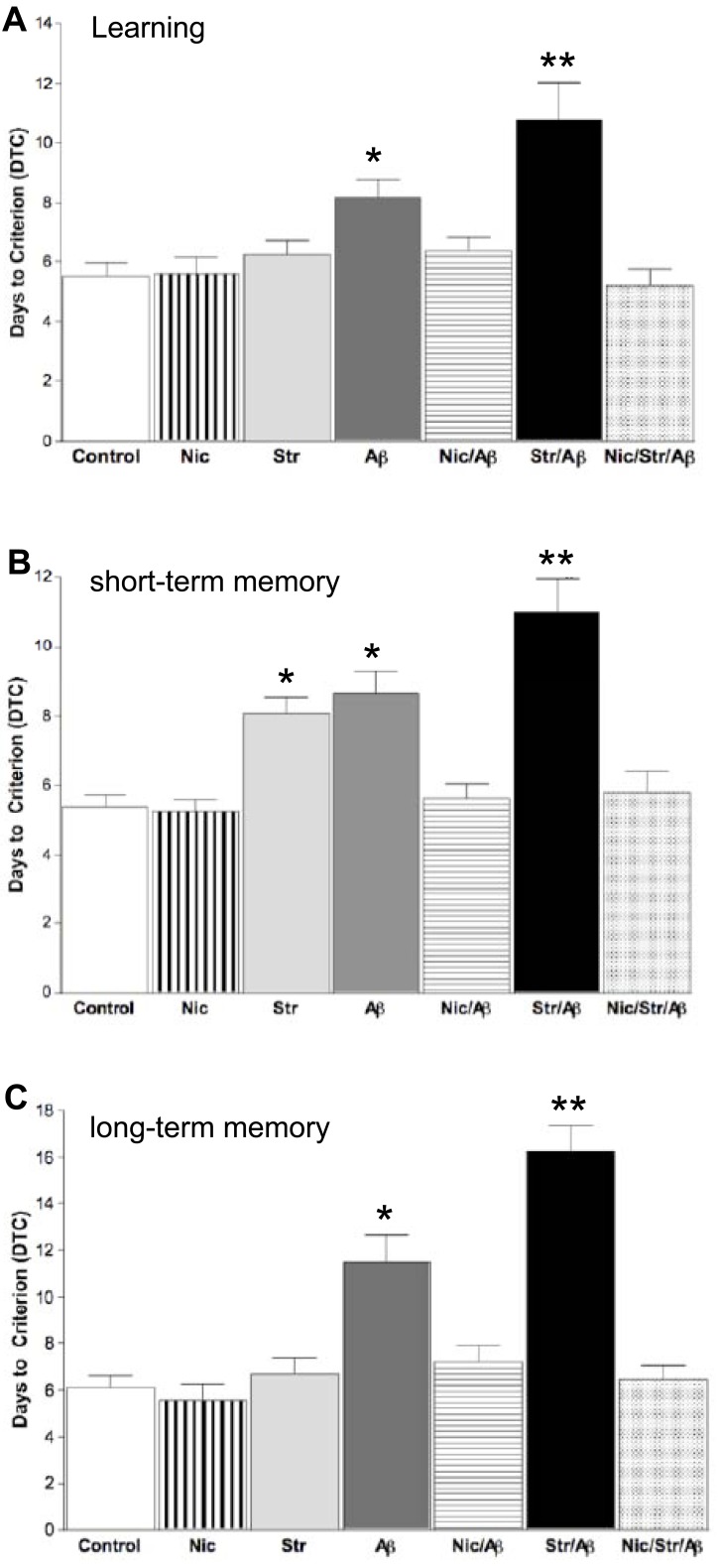
Days to criterion (DTC) values; criterion is reached when a rat makes a maximum of one error per three consecutive days of testing in the RAWM. Chronic nicotine treatment prevents stress-exacerbated impairment of learning, short-term, and long-term memory in Aβ rats. (**A**) Memory function, measured as reaching DTC, indicated that the combination of stress and Aβ impaired learning more than stress or Aβ, alone, thus requiring stress/Aβ rats to be trained for a minimum of 17 days in the RAWM in order to reach criterion. Neither nicotine, nor stress, alone, affects acquisition or long-term memory performance (**C**). Chronic nicotine treatment significantly prevents stress exacerbated Aβ-impairment of short-term (**B**) and long-term memory (**C**). All values are mean ± S.E.M. (n=12-15 rats/group); *p<0.05 compared to control and nicotine-treated groups; **p<0.05 compared to all groups. Modified from references [18, 20].

**Fig. (2):**
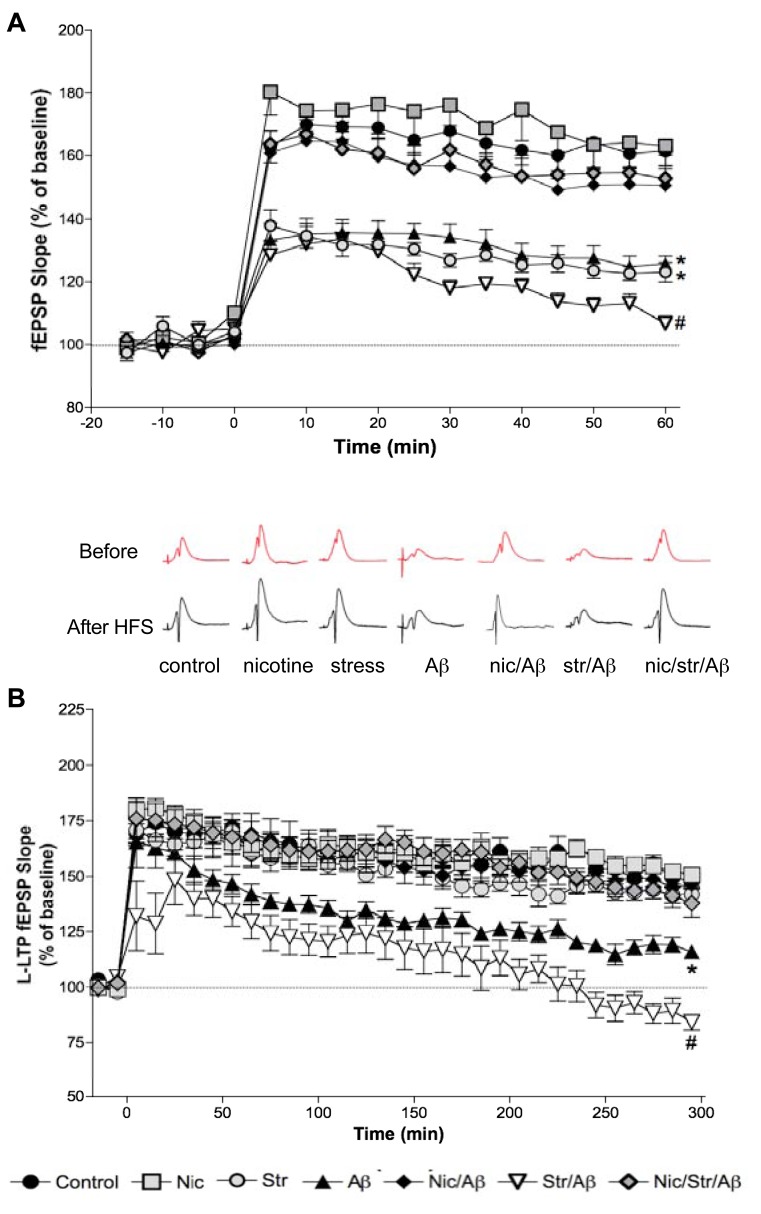
Hippocampal long-term potentiation (LTP) of the CA1 region evoked by repetitive stimulation (applied at t=0) of the Schaffer collaterals/commissural pathway and measured as increases in the slope of fEPSP in urethane-anesthetized rats. (**A**) Early phase (E-LTP), evoked by high frequency stimulation (HFS), was significantly decreased in stress and/or Aβ groups. Chronic nicotine treatment significantly prevented stress-induced E-LTP suppression in Aβ rats. (**B**) Late-phase (L-LTP), evoked by multiple high frequency stimulation (MHFS), was significantly decreased in the Aβ and stress/Aβ groups. Chronic nicotine treatment prevented the effects of both stress and/or Aβ. Values are mean ± S.E.M. from 5-8 rats (^*^p<0.05 compared to control animals; #p<0.05 compared to all animals). Insets are traces from representative experiments. Modified from references [18, 19].

**Fig. (3):**
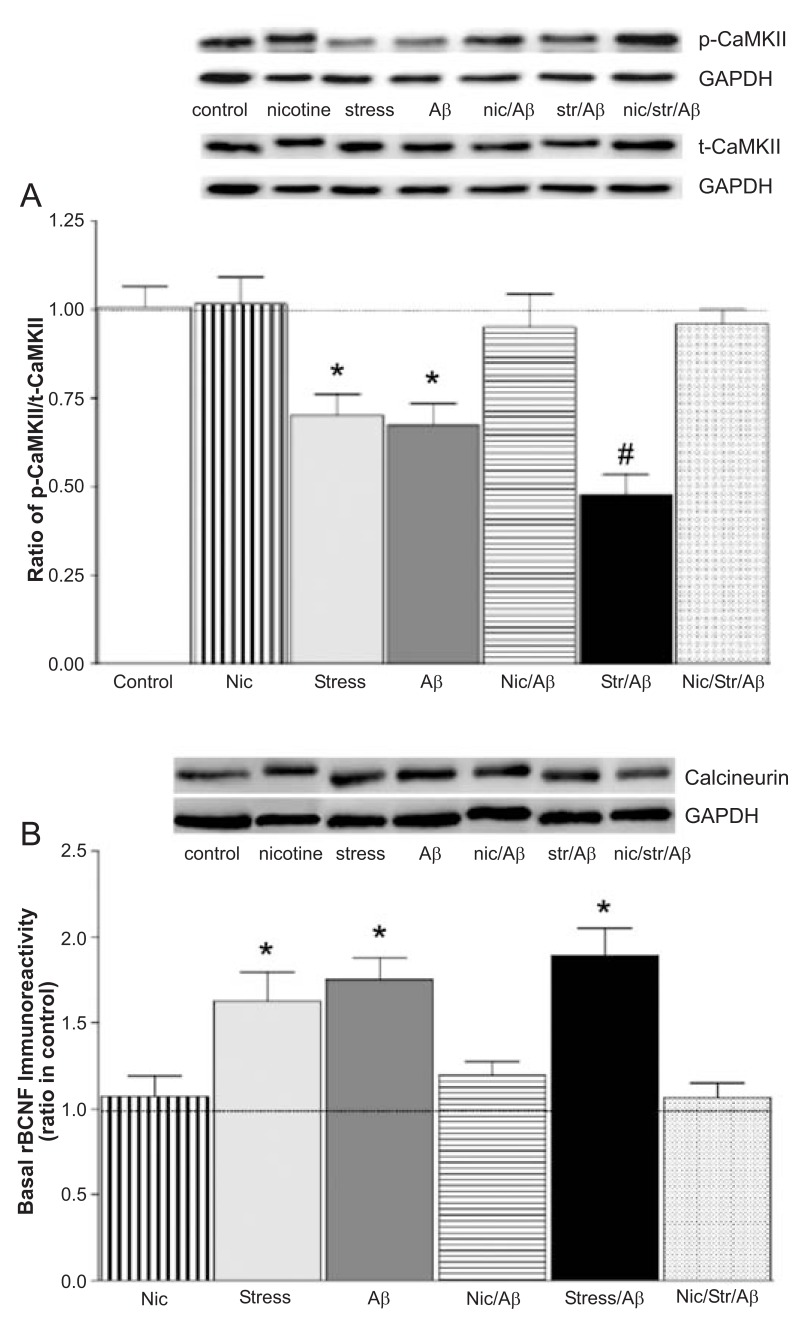
(**A**) Ratio of basal levels of phosphorylated-calcium-calmodulin-dependent protein kinase II (p-CaMKII) to total (t)-CaMKII in CA1. Values are mean ± S.E.M. from 5-7 rats/group (^*^p<0.05 compared to control and nicotine-treated animals; #p<0.05 compared to all groups). (**B**) Basal levels of calcineurin in area CA1. Nicotine prevents the adverse effect of chronic stress on Aβ-induced increases in basal calcineurin levels in hippocampal homogenates. Values are mean ± S.E.M. from 5-7 rats (^*^p<0.05 compared to control and nicotine-treated animals (nicotine, nicotine/Aβ, nicotine/stress/Aβ). Insets: representative immunoblot images. Modified from references [18, 19].

**Fig. (4):**
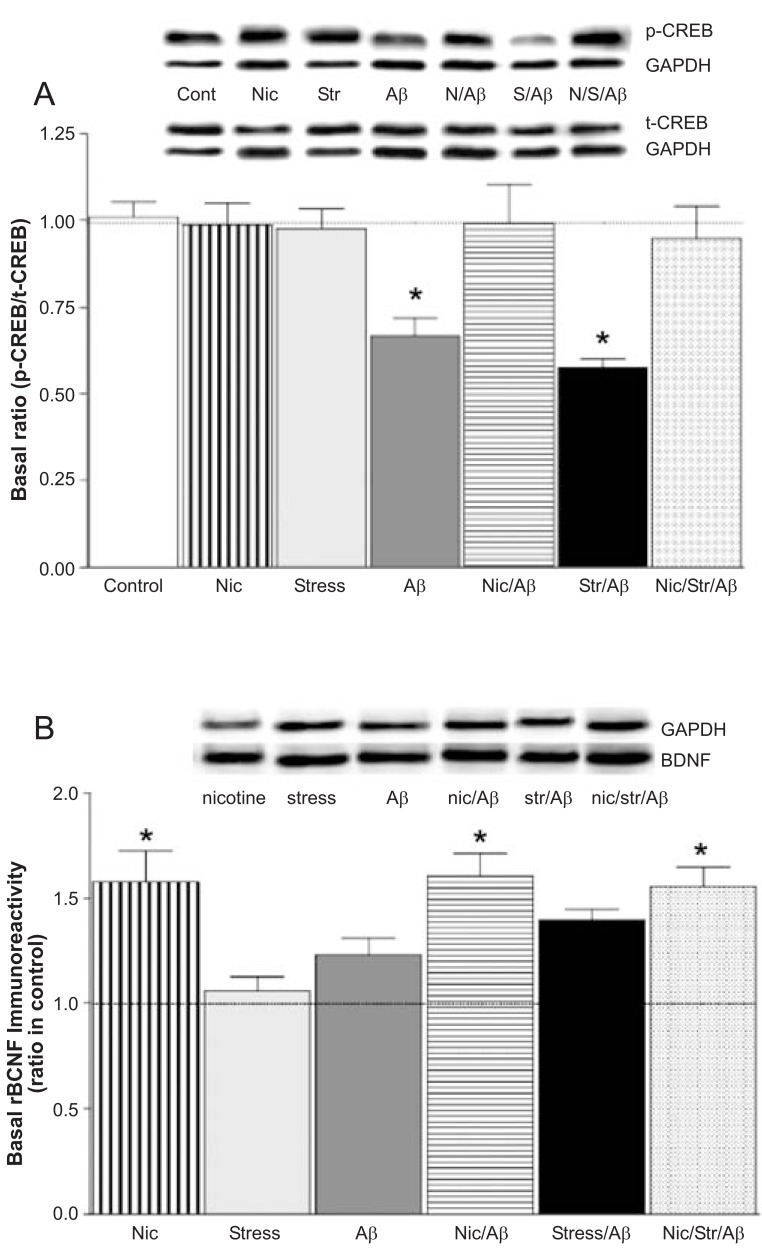
(**A**) Ratio of basal levels of phosphorylated-cyclic AMP response element binding (p-CREB) to total (t)-CREB in area CA1. Values are mean ± S.E.M. from 5-7 rats/group (^*^p<0.05 compared to control and nicotine-treated animals). (**B**) Basal levels of brainderived neurotrophic factor (BDNF) in area CA1. Animals were treated with (-) nicotine or saline for 6 weeks. Groups treated with nicotine showed increased levels of BDNF (Values are mean ± S.E.M.; n=5-7 rats/group; ^*^p<0.01 compared to control animals. Modified from reference [20].

**Table 1.** Summary of the Effects of Nicotine and/or Stress on the Basal Levels of Essential Signalling Molecules in CA1 Area of the Hippocampus in Aβ-Treated Rats Compared to Control. ⇓Decreased, ⇑ Increased, ⇔ No Change

	Nicotine	Stress	Ab	Nic/Ab	Str/Ab	Nic/Str/Ab
p-CaMKII	⇔	⇓	⇓	⇔	⇓	⇔
t-CaMKII	⇔	⇔	⇔	⇔	⇔	⇔
Calcineurin	⇔	⇑	⇑	⇔	⇑	⇔
BDNF	⇑	⇔	⇑	⇑	⇑	⇑
p-CREB	⇔	⇔	⇓	⇔	⇓	⇔
t-CREB	⇔	⇔	⇔	⇔	⇔	⇔
CaMKIV	⇔	⇔	⇓	⇔	⇓	⇔

## References

[1] Castellani RJ, Lee HG, Zhu X, Perry G, Smith MA (2008). Alzheimer disease pathology as a host response. J. Neuropathol. Exp. Neurol.

[2] Tanzi RE, Bertram L (2005). Twenty years of the Alzheimer's disease amyloid hypothesis, a genetic perspective. Cell.

[3] Selkoe DJ (2002). Alzheimer's disease is a synaptic failure. Science.

[4] Selkoe DJ (2004). Alzheimer disease, mechanistic understanding predicts novel therapies. Ann. Intern. Med.

[5] Williamson J, Goldman J, Marder KS (2009). Genetic aspects of Alzheimer disease. Neurologist.

[6] Shankar GM, Li S, Mehta TH, Garcia-Munoz A, Shepardson NE, Smith I, Brett FM, Farrell MA, Rowan MJ, Lemere CA, Regan CM, Walsh DM, Sabatini BL, Selkoe DJ (2008). Amyloid-beta protein dimers isolated directly from Alzheimer's brains impair synaptic plasticity and memory. Nat. Med.

[7] Sakono M, Zako T (2010). Amyloid oligomers, formation and toxicity of Abeta oligomers. FEBS J.

[8] Mesulam MM (1999). Neuroplasticity failure in Alzheimer's disease, bridging the gap between plaques and tangles. Neuron.

[9] Aleisa AM, Alzoubi KH, Gerges NZ, Alkadhi KA (2006). Chronic psychosocial stress-induced impairment of hippocampal LTP: possible role of BDNF. Neurobiol. Dis.

[10] Garcia R (2001). Stress hippocampal plasticity and spatial learning. Synapse.

[11] Gerges NZ, Aleisa AM, Schwarz LA, Alkadhi KA (2004). Reduced basal CaMKII levels in hippocampal CA1 region, possible cause of stress-induced impairment of LTP in chronically stressed rats. Hippocampus.

[12] McEwen BS, Sapolsky RM (1995). Stress and cognitive function. Curr. Opin. Neurobiol.

[13] Miller DB, O'Callaghan JP (2002). Neuroendocrine aspects of the response to stress. Metabolism.

[14] Sandi C, Pinelo-Nava MT (2007). Stress and memory, behavioral effects and neurobiological mechanisms. Neural Plast.

[15] Walker E, Mittal V, Tessner K (2008). Stress and the hypothalamic pituitary adrenal axis in the developmental course of schizophrenia. Annu. Rev. Clin. Psychol.

[16] Whitworth JA, Mangos GJ, Kelly JJ (2000). Cushing cortisol and cardiovascular disease. Hypertension.

[17] Gerges NZ, Alzoubi KH, Park CR, Diamond DM, Alkadhi KA (2004). Adverse effect of the combination of hypothyroidism and chronic psychosocial stress on hippocampus-dependent memory in rats. Behav. Brain Res.

[18] Srivareerat M, Tran TT, Alzoubi KH, Alkadhi KA (2009). Chronic psychosocial stress exacerbates impairment of cognition and long-term potentiation in beta-amyloid rat model of alzheimer's disease. Biol. Psychiatry.

[19] Srivareerat M, Tran TT, Salim S, Aleisa AM, Alkadhi KA (2011). Chronic nicotine restores normal Aß levels and prevents short-term memory and E-LTP impairment in Aß rat model of Alzheimer’s disease. Neurobiol. Aging.

[20] Alkadhi KA, Srivareerat M, Tran TT (2010). Intensification of long-term memory deficit by chronic stress and prevention by nicotine in a rat model of Alzheimer’s disease. Mol. Cell. Neurosci.

[21] Alkadhi KA, Srivareerat M, Tran TT (2011). Chronic psychosocial stress exacerbates impairment of synaptic plasticity in ?-Amyloid rat model of Alzheimer’s disease: Prevention by nicotine. Curr. Alz. Res.

[22] Armanini D, Vecchio F, Basso A, Milone FF, Simoncini M, Fiore C, Mattarello MJ, Sartorato P, Karbowiak I (2003). Alzheimer's disease: pathophysiological implications of measurement of plasma cortisol, plasma dehydroepiandrosterone sulfate, and lymphocytic corticosteroid receptors. Endocrine.

[23] Csernansky JG, Dong H, Fagan AM, Wang L, Xiong C, Holtzman DM, Morris JC (2006). Plasma cortisol and progression of dementia in subjects with Alzheimer-type dementia. Am. J. Psychiatry.

[24] de Bruin V, Vieira MC, Rocha MN, Viana GS (2002). Cortisol and dehydroepiandosterone sulfate plasma levels and their relationship to aging cognitive function and dementia. Brain Cogn.

[25] Hartmann A, Veldhuis JD, Deuschle M, Standhardt H, Heuser I (1997). Twenty-four hour cortisol release profiles in patients with Alzheimer's and Parkinson's disease compared to normal controls, ultradian secretary pulsatility and diurnal variation. Neurobiol. Aging.

[26] Dong H, Goico B, Martin M, Csernansky CA, Bertchume A, Csernansky JG (2004). Modulation of hippocampal cell proliferation memory and amyloid plaque deposition in APPsw (Tg2576) mutant mice by isolation stress. Neuroscience.

[27] Landfield PW, Blalock EM, Chen KC, Porter NM (2007). A new glucocorticoid hypothesis of brain aging, implications for Alzheimer's disease. Curr. Alzheimer Res.

[28] Sauro MD, Jorgensen RS, Pedlow CT (2003). Stress glucocorticoids and memory, a meta-analytic review. Stress.

[29] Wilson RS, Evans DA, Bienias JL, Mendes de Leon CF, Schneider JA, Bennett DA (2003). Proneness to psychological distress is associated with risk of Alzheimer's disease. Neurology.

[30] Wilson RS, Schneider JA, Boyle PA, Arnold SE, Tang Y, Bennett DA (2007). Chronic distress and incidence of mild cognitive impairment. Neurology.

[31] Elgh E, Lindqvist AA, Stot A, Fagerlund M, Eriksson S, Olsson T, Näsman B (2006). Cognitive dysfunction hippocampal atrophy and glucocorticoid feedback in Alzheimer's disease. Biol. Psychiatry.

[32] Budas G, Coughlan CM, Seckl JR, Breen KC (1999). The effect of corticosteroids on amyloid beta precursor protein/amyloid precursor-like protein expression and processing *in vivo*. Neurosci. Lett.

[33] Islam A, Kalaria RN, Winblad B, Adem A (1998). Enhanced localization of amyloid beta precursor protein in the rat hippocampus following long-term adrenalectomy. Brain Res.

[34] Kim JJ, Yoon KS (1998). Stress, metaplastic effects in the hippocampus. Trends Neurosci.

[35] Pavlides C, Watanabe Y, Magarinos AM, McEwen BS (1995). Opposing roles of type I and type II adrenal steroid receptors in hippocampal long-term potentiation. Neuroscience.

[36] Tsigos C, Chrousos GP (2002). Hypothalamic-pituitary-adrenal axis neuroendocrine factors and stress. J. Psychosom. Res.

[37] McEwen BS (1999). Stress and hippocampal plasticity. Annu. Rev. Neurosci.

[38] Brenner DE, Kukull WA, van Belle G, Bowen JD, McCormick WC, Teri L, Larson EB (1993). Relationship between cigarette smoking and Alzheimer's disease in a population-based case-control study. Neurology.

[39] Hillier V, Salib E (1997). A case-control study of smoking and Alzheimer's disease. Intl. J. Geriat. Psychiatry.

[40] Potter A, Corwin J, Lang J, Piasecki M, Lenox R, Newhouse PA (1999). Acute effects of the selective cholinergic channel activator (nicotinic agonist) ABT-418 in Alzheimer's disease. Psychopharmacology.

[41] Ulrich J, Johannson-Locher G, Seiler WO, Stahelin HB (1997). Does smoking protect from Alzheimer's disease? Alzheimer-type changes in 301 unselected brains from patients with known smoking history. Actaneuropathologica.

[42] Nooyens AC, van Gelder BM, Verschuren WM (2008). Smoking and cognitive decline among middle-aged men and women, the Doetinchem Cohort Study. Am. J. Public Health.

[43] Swan GE, Lessov-Schlaggar CN (2007). The effects of tobacco smoke and nicotine on cognition and the brain. Neuropsychol. Rev.

[44] Emilien G, Beyreuther K, Masters CL, Maloteaux JM (2000). Prospects for pharmacological intervention in Alzheimer disease. Arch. Neurol.

[45] Maurice T, Lockhart BP, Privat A (1996). Amnesia induced in mice by centrally administered beta-amyloid peptides involves cholinergic dysfunction. Brain Res.

[46] Takita M, Taniguchi T, Zhu J, Piao HL, Tsai TY, Muramatsu I (1999). Effects of chronic treatment with (+)-nicotine on the stress-induced hypertension and downregulation of central nicotinic receptors in rats, comparative study with (-)-nicotine. Gen. Pharmacol.

[47] Borchelt DR, Thinakaran G, Eckman CB, Lee MK, Davenport F, Ratovitsky T, Prada CM, Kim G, Seekins S, Yager D, Slunt HH, Wang R, Seeger M, Levey AI, Gandy SE, Copeland NG, Jenkins NA, Price DL, Younkin SG, Sisodia SS (1996). Familial Alzheimer's disease-linked presenilin 1 variants elevate Abeta1-42/1-40 ratio *in vitro* and *in vivo*. Neuron.

[48] Duff K, Eckman C, Zehr C, Yu X, Prada CM, Perez-tur J, Hutton M, Buee L, Harigaya Y, Yager D, Morgan D, Gordon MN, Holcomb L, Refolo L, Zenk B, Hardy J, Younkin S (1996). Increased amyloid-beta42(43) in brains of mice expressing mutant presenilin 1. Nature.

[49] Kammesheidt A, Boyce FM, Spanoyannis AF, Cummings BJ, Ortegon M, Cotman C, Vaught JL, Neve RL (1992). Deposition of beta/A4 immunoreactivity and neuronal pathology in transgenic mice expressing the carboxyl-terminal fragment of the Alzheimer amyloid precursor in the brain. Proc. Natl. Acad. Sci. USA.

[50] LaFerla FM, Tinkle BT, Bieberich CJ, Haudenschild CC, Jay G (1995). The Alzheimer's A beta peptide induces neurodegeneration and apoptotic cell death in transgenic mice. Nat. Genet.

[51] Quon D, Wang Y, Catalano R, Scardina JM, Murakami K, Cordell B (1991). Formation of beta-amyloid protein deposits in brains of transgenic mice. Nature.

[52] Ashe KH (2001). Learning and memory in transgenic mice modeling Alzheimer's disease. Learn. Mem.

[53] Chapman PF, Falinska AM, Knevett SG, Ramsay MF (2001). Genes, models and Alzheimer's disease. Trends Genet.

[54] Janus C, Chishti MA, Westaway D (2000). Transgenic mouse models of Alzheimer's disease. Biochim. Biophys. Acta.

[55] Richardson JA, Burns DK (2002). Mouse models of Alzheimer's disease, a quest for plaques and tangles. Ilar. J.

[56] Oddo S, Caccamo A, Smith IF, Green KN, LaFerla FM (2006). A dynamic relationshipbetween intracellular and extracellular pools of Abeta. Am. J. Pathol.

[57] Oddo S, LaFerla FM (2006). The role of nicotinic acetylcholine receptors in Alzheimer’sdisease. J. Physiol. (Paris).

[58] Stephan A, Phillips AG (2005). A case for a non-transgenic animal model of Alzheimer's disease. Genes Brain Behav.

[59] Nabeshima T, Itoh A (1998). Toxicity of beta-amyloid peptide. J. Toxicol. Sci.

[60] Pepeu G, Giovannelli L, Casamenti F, Scali C, Bartolini L (1996). Amyloid beta-peptides injection into the cholinergic nuclei, morphological neurochemical and behavioral effects. Prog. Brain Res.

[61] Seubert P, Vigo-Pelfrey C, Esch F, Lee M, Dovey H, Davis D, Sinha S, Schiossmacher M, Whaley J, Swindlehurst C, McCormack R, Wolfert R, Selkoe D, Lieberburg I, Schenk D (1992). Isolation and quantification of soluble Alzheimer's beta-peptide from biological fluids. Nature.

[62] Shoji M, Golde T E, Ghiso J, Cheung TT, Estus S, Shaffer LM, Cai XD, McKay DM, Tintner R, Frangione B, Younkin SG (1992). Production of the Alzheimer amyloid beta protein by normal proteolytic processing. Science.

[63] Yang L, Wang B, Long C, Wu G, Zheng H (2007). Increased asynchronous release and aberrant calcium channel activation in amyloid precursor protein deficient neuromuscular synapses. Neuroscience.

[64] Jarrett JT, Lansbury  PT (1992). Amyloid fibril formation requires a chemically discriminating nucleation event, studies of an amyloidogenic sequence from the bacterial protein OsmB. Biochemistry.

[65] Jarrett JT, Lansbury PT (1993). Seeding "one-dimensional crystallization" of amyloid, a pathogenic mechanism in Alzheimer's disease and scrapie?. Cell.

[66] Snyder SW, Ladror US, Wade WS, Wang GT, Barrett LW, Matayoshi ED, Huffaker HJ, Krafft GA, Holzman TF (1994). Amyloid-beta aggregation, selective inhibition of aggregation in mixtures of amyloid with different chain lengths. Biophys. J.

[67] Alamed J, Wilcock DM, Diamond DM, Gordon MN, Morgan D (2006). Two-day radial-arm water maze learning and memory task, robust resolution of amyloid-related memory deficits in transgenic mice. Nat. Protoc.

[68] Buresova O, Bures J, Oitzl MS, Zahalka A (1985). Radial maze in the water tank: an aversively motivated spatial working memory task. Physiol. Behav.

[69] Diamond DM, Park CR Heman, K.L. Rose GM (1999). Exposing rats to a predator impairs spatial working memory in the radial arm water maze. Hippocampus.

[70] Tran TT, Srivareerat M, Alkadhi KA (2010). Chronic psychosocial stress accelerates impairment of long-term memory and late-phase long-term potentiation in an at-risk model of Alzheimer’s disease. Hippocampus.

[71] Tran TT, Srivareerat M, Alkadhi KA (2010). Chronic psychosocial stress triggers cognitive impairment in a novel at-risk model of Alzheimer’s disease. Neurobiol. Dis.

[72] Alzoubi KH, Aleisa AM, Alkadhi KA (2008). Effect of chronic stress or nicotine on hypothyroidism–induced enhancement of LTD: electrophysiological and molecular studies. Neurobiol. Dis.

[73] Aleisa AM, Alzoubi KH, Gerges NZ, Alkadhi KA (2006). Nicotine blocks stress-induced impairment of spatial memory and long-term potentiation of the hippocampal CA1 region. Int. J. Neuropsychopharmacol.

[74] Bliss TV, Collingridge GL (1993). A synaptic model of memory: long-term potentiation in the hippocampus. Nature.

[75] Aleisa A M, Alzoubi K H, Alkadhi K A (2006). Nicotine prevents stress-induced enhancement of LTD: electrophysiological and molecular studies. J. Neurosci. Res.

[76] Fukunaga K, Stoppini L, Miyamoto E, Muller D (1993). Long-term potentiation is associated with an increased activity of Ca^2+^/calmodulin-dependent protein kinase II. J. Biol. Chem.

[77] Malenka RC, Nicoll RA (1999). Long-term potentiation--a decade of progress?. Science.

[78] Malinow R, Schulman H, Tsien RW (1989). Inhibition of postsynaptic PKC or CaMKII blocks induction but not expression of LTP. Science.

[79] Fornasiero EF, Bonanomi D, Benfenati F, Valtorta F (2010). The role of synapsins in neuronal development. Cell. Mol. Life Sci.

[80] Nayak AS, Moore CI, Browning MD (1996). Ca^2+^/calmodulin-dependent protein kinase II phosphorylation of the presynaptic protein synapsin I is persistently increased during long-term potentiation. Proc. Natl. Acad. Sci. USA.

[81] Lisman J, Schulman H, Cline H (2002). The molecular basis of CaMKII function in synaptic and behavioural memory. Nat. Rev. Neurosci.

[82] Taglialatela G, Hogan D, Zhang WR, Dineley KT (2009). Intermediate- and long-term recognition memory deficits in Tg2576 mice are reversed with acute calcineurin inhibition. Behav. Brain Res.

[83] Alberini CM (2009). Transcription factors in long-term memory and synaptic plasticity. Physiol. Rev.

[84] Bourtchuladze R, Frenguelli B, Blendy J, Cioffi D, Schutz G, Silva AJ (1994). Deficient long-term memory in mice with a targeted mutation of the cAMP-responsive element-binding protein. Cell.

[85] Tong L, Thornton PL, Balazs R, Cotman CW (2001). Beta -amyloid-(1-42) impairs activity-dependent cAMP-response element-binding protein signaling in neurons at concentrations in which cell survival Is not compromised. J. Biol. Chem.

[86] Tokuda M, Ahmed BY, Lu YF, Matsui H, Miyamoto O, Yamaguchi F, Konishi R, Hatase O (1997). Involvement of calmodulin-dependent protein kinases-I and -IV in long-term potentiation. Brain Res.

[87] Durany N, Michel T, Kurt J, Cruz-Sanchez FF, Cervas-Navarro J, Riederer P (2000). Brain-derived neurotrophic factor and neurotrophin-3 levels in Alzheimer's disease brains. Int. J. Dev. Neurosci.

[88] Alonso M, Vianna MR, Depino AM, Mello e Souza T, Pereira P, Szapiro G, Viola H, Pitossi F, Izquierdo I, Medina JH (2002). BDNF-triggered events in the rat hippocampus are required for both short- and long-term memory formation. Hippocampus.

[89] Zuccato C, Cattaneo E (2009). Brain-derived neurotrophic factor in neurodegenerative diseases. Nat. Rev. Neurol.

[90] Hellstrom-Lindahl E, Court J, Keverne J, Svedberg M, Lee M, Marutle A, Thomas A, Perry E, Bednar I, Nordberg A (2004). Nicotine reduces A beta in the brain and cerebral vessels of APPsw mice. Eur. J. Neurosci.

[91] Barde YA (1989). Trophic factors and neuronal survival. Neuron.

[92] Hock C, Heese K, Muller-Spahn F, Hulette C, Rosenberg C, Otten U (1998). Decreased trkA neurotrophin receptor expression in the parietal cortex of patients with Alzheimer's disease. Neurosci. Lett.

[93] Townsend M, Mehta T, Selkoe DJ (2007). Soluble Abeta inhibits specific signal transduction cascades common to the insulin receptor pathway. J. Biol. Chem.

[94] Zhao D, Watson JB, Xie CW (2004). Amyloid beta prevents activation of calcium/calmodulin-dependent protein kinase II and AMPA receptor phosphorylation during hippocampal long-term potentiation. J. Neurophysiol.

[95] Conrad CD, Lupien SJ, McEwen BS (1999). Support for a bimodal role for type II adrenal steroid receptors in spatial memory. Neurobiol. Learn. Mem.

[96] Liang Z, Liu F, Grundke-Iqbal I, Iqbal K, Gong CX (2007). Down-regulation of cAMP-dependent protein kinase by over-activated calpain in Alzheimer disease brain. J. Neurochem.

[97] Liu F, Grundke-Iqbal I, Iqbal K, Oda Y, Tomizawa K, Gong CX (2005). Truncation and activation of calcineurin A by calpain I in Alzheimer disease brain. J. Biol. Chem.

[98] Lindvall O, Ernfors P, Bengzon J, Kokaia Z, Smith ML, Siesjo BK, Persson H (1992). Differential regulation of mRNAs for nerve growth factor brain-derived neurotrophic factor and neurotrophin 3 in the adult rat brain following cerebral ischemia and hypoglycemic coma. Proc. Natl. Acad. Sci. USA.

[99] Todaro L, Puricelli L, Gioseffi H, Guadalupe Pallotta M, Lastiri J, Bal de Kier Joffé E, Varela M, Sacerdote de Lustig E (2004). Neural cell adhesion molecule in human serum. Increased levels in dementia of the Alzheimer type. Neurobiol. Dis.

[100] Sandi C (2004). Stress cognitive impairment and cell adhesion molecules. Nat. Rev. Neurosci.

[101] Cordero MI, Rodriguez JJ, Davies HA, Peddie CJ, Sandi C, Stewart MG (2005). Chronic restraint stress down-regulates amygdaloid expression of polysialylated neural cell adhesion molecule. Neuroscience.

[102] Catania C, Sotiropoulos I, Silva R, Onofri C, Breen KC, Sousa N, Almeida OF (2009). The amyloidogenic potential and behavioral correlates of stress. Mol. Psychiatry.

[103] Lee KW, Kim JB, Seo JS, Kim TK, Im JY, Baek IS, Kim KS, Lee JK, Han PL (2009). Behavioral stress accelerates plaque pathogenesis in the brain of Tg2576 mice *via* generation of metabolic oxidative stress. J. Neurochem.

[104] Salomon AR, Marcinowski KJ, Friedland RP, Zagorski MG (1996). Nicotine inhibits amyloid formation by the beta-peptide. Biochemistry.

[105] Dickerson TJ, Janda KD (2003). Glycation of the amyloid beta-protein by a nicotine metabolite, a fortuitous chemical dynamic between smoking and Alzheimer's disease. Proc. Natl. Acad. Sci. USA.

[106] Zeng H, Zhang Y, Peng L, Shao H, Menon NK, Yang J, Salomon AR, Freidland RP, Zagorski MG (2001). Nicotine and amyloid formation. Biol. Psychiatry.

[107] Hilbich C, Kisters-Woike B, Reed J, Masters CL, Beyreuther K (1991). Aggregation and secondary structure of synthetic amyloid beta A4 peptides of Alzheimer's disease. J. Mol. Biol.

[108] Moore SA, Huckerby TN, Gibson GL, Fullwood NJ, Turnbull S, Tabner BJ, El-Agnaf OM, Allsop D (2004). Both the D-(+) and L-(-) enantiomers of nicotine inhibit Abeta aggregation and cytotoxicity. Biochemistry.

[109] Lahiri DK, Utsuki T, Chen D, Farlow MR, Shoaib M, Ingram DK, Greig NH (2002). Nicotine reduces the secretion of Alzheimer's beta-amyloid precursor protein containing beta-amyloid peptide in the rat without altering synaptic proteins. Ann. N. Y. Acad. Sci.

[110] Utsuki T, Shoaib M, Holloway HW, Ingram DK, Wallace WC, Haroutunian V, Sambamurti K, Lahiri DK, Greig NH (2002). Nicotine lowers the secretion of the Alzheimer's amyloid beta-protein precursor that contains amyloid beta-peptide in rat. J. Alzheimers Dis.

[111] Belluardo N, Blum M, Mudo G, Andbjer B, Fuxe K (1998). Acute intermittent nicotine treatment produces regional increases of basic fibroblast growth factor messenger RNA and protein in the tel- and diencephalon of the rat. Neuroscience.

[112] Maggio R, Riva M, Vaglini F, Fornai F, Racagni G, Corsini GU (1997). Striatal increase of neurotrophic factors as a mechanism of nicotine protection in experimental parkinsonism. J. Neural Transm.

[113] Shimohama S, Akaike A, Kimura J (1996). Nicotine-induced protection against glutamate cytotoxicity Nicotinic cholinergic receptor-mediated inhibition of nitric oxide formation. Ann. N. Y. Acad. Sci.

[114] Marin P, Hamon B, Glowinski J, Premont J (1997). Nicotine-induced inhibition of neuronal phospholipase A2. J. Pharmacol. Exp. Ther.

[115] Rosas-Ballina M, Ochani M, Parrish WR, Ochani K, Harris YT, Huston JM, Chavan S, Tracey KJ (2008). Splenic nerve is required for cholinergic antiinflammatory pathway control of TNF in endotoxemia. Proc. Natl. Acad. Sci. USA.

[116] Rosas-Ballina M, Goldstein RS, Gallowitsch-Puerta M, Yang L, Valdés-Ferrer SI, Patel NB, Chavan S, Al-Abed Y, Yang H, Tracey KJ (2009). The selective alpha7 agonist GTS-21 attenuates cytokine production in human whole blood and human monocytes activated by ligands for TLR2 TLR3 TLR4 TLR9 and RAGE. Mol. Med.

[117] Saeed RW, Varma S, Peng-Nemeroff T, Sherry B, Balakhaneh D, Huston J, Tracey KJ, Al-Abed Y, Metz CN (2005). Cholinergic stimulation blocks endothelial cell activation and leukocyte recruitment during inflammation. J. Exp. Med.

[118] vanMaanen MA, Lebre MC, van der Poll T, LaRosa GJ, Elbaum D, Vervoordeldonk MJ, Tak PP (2009). Stimulation of nicotinic acetylcholine receptors attenuates collagen-induced arthritis in mice. Arthritis Rheum.

